# Randomized clinical trial: Nucleos(t)ide analogues improved survival of CHB-related HCC patients *via* reducing severity and progression of malignancy

**DOI:** 10.18632/oncotarget.10155

**Published:** 2016-06-18

**Authors:** Yun Wang, Xiaogang Xiang, Liwen Chen, Zhujun Cao, Rebecca Bao, Huijuan Zhou, Weiliang Tang, Jie Lu, Lanyi Lin, Qing Xie, Shisan Bao, Hui Wang

**Affiliations:** ^1^ Department of Infectious Diseases and Hepatology, Ruijin Hospital, Shanghai Jiaotong University School of Medicine, Shanghai, China; ^2^ Discipline of Anatomy and Histology F13, The University of Sydney, New South Wales, Australia; ^3^ Discipline of Pathology, School of Medical Sciences and Bosch Institute, The University of Sydney, New South Wales, Australia

**Keywords:** nucleos(t)ide analogues, survival, chronic hepatitis B, hepatocellular carcinoma

## Abstract

**Background:**

The influence of nucleos(t)ide analogues (NAs) to treat Chronic hepatitis B (CHB) related hepatocellular carcinoma (HCC) remains to be explored.

**Aim:**

To investigate if NAs reduce the severity and progression of CHB-related HCC.

**Results:**

Among 532 patients, there were 118 or 414 CHB-related HCC with or without NAs therapy, respectively. BCLC scores, serum level of ALT/AST and HBV DNA were compared. During follow-up, the survival period of CHB-related HCC patients with sustained NAs is significantly longer than that with NAs post-HCC and NAs naïve (*p* < 0.05). Factors significantly associated with the poor overall survival of CHB-related HCC include BCLC scores (hazard ratio, 1.84 [95% confidence interval, 1.57−2.15], *p* < 0.001), NAs post-HCC or NAs naïve (1.33 [1.07−1.65], *p* < 0.01), serum AST ≥ 40 IU/L (1.48 [1.03−2.12], *p* < 0.05) and HBV DNA ≥ 10^4^ copies/ml (1.36 [1.01−1.83], *p* < 0.001).

**Methods:**

Outcomes of 532 CHB-related HCC patients with/without NAs were investigated. Overall survival of CHB-related HCC patients, NAs naïve (*n* = 156), NAs received post-HCC (*n* = 258) and NAs sustained (*n* = 118) were determined.

**Conclusions:**

NAs reduced severity of CHB-related HCC patients. Sustained NAs is an important factor associated with the extended survival of CHB-related HCC patients.

## INTRODUCTION

Hepatocellular carcinoma (HCC) is one of the most common malignancies with high morbidity and mortality worldwide [1], particularly in China [2]. The number of Chinese HCC patients account for > 50% of HCC-associated death at the global level [1, 3, 4]. Chronic hepatitis B (CHB) infection is the most highly linked with HCC worldwide [5], although other hepatic injuries also contribute to development of HCC, including HCV infection, alcoholic or fatty liver disease, or smoking [6]. CHB-related HCC patients accounted for ~80% in HCC patients in China [7], due to a high prevalence of HBV infection [8], which has attracted extensive attention in medical practitioners and policy makers in China.

The viral load in circulating serum of CHB patients is a key predictor of cirrhosis risk, as it has been demonstrated that there is close correlation between viral load and progression to cirrhosis [9]. Persistent replication of HBV and high plasma HBV DNA concentrations in CHB patients contributes to uncontrollable progression of cirrhosis, eventually resulting in CHB-related HCC and liver-related death [9–11]. It has been demonstrated that high level of HBV viral load in CHB patients is a critical risk factor for the progression of HCC [12]. Thus, treatment for CHB aims to suppress viral proliferation, leading to reduce liver fibrosis and preventing hepatic decomposition and/or HCC [13]. It has been demonstrated that nucleos(t) ide analogues (NAs) are highly effective in the suppression circulating viral loads, but rarely eliminate the virus over the last ten years [14]. Single-hospital based studies found that long-term NAs treatment reduces substantially the HCC risk in CHB patients, compare to that of NA-naive patients [14–17]. However, more than 70% of HCCs patients were not eligible for surgical resection, due to tumor progression and/or underlying advanced liver cirrhosis at the time of diagnosis of HCC [5].

It has been reported that NAs improve overall survival of CHB-related HCC patients treated with sorafenib (chemotherapy), especially in patients with BCLC stage C and high HBV-DNA level [18]. Furthermore, NAs also extend the survival time in CHB-related HCC patients following curative resection [18, 19]. Early anti-viral intervention reduces HCC development in CHB patients, as well as minimizes HCC recurrence following resection HCC in combination with adjuvant in these patients with a high HBV-DNA level [20]. Therefore the data above suggests that there is significant benefit of NAs in reducing progression of HCC in CHB patients.

However, there are still a relatively large number of CHB patients developed HCC, despite NAs reduce the risk of the overall mortality of HCC. The aim of the current study was to explore if there is reverse correlation between NAs and severity of HCC, including differentiation and progression in CHB patients.

## RESULTS

### The characteristics of 532 patients

Total 532 final identified CHB-related HCC patients were divided into NAs treated (*n* = 118) and NA naïve (*n* = 414) groups (Table 1). The mean age of NAs treated or NAs naïve group was 55 or 52, respectively, at baseline. Male versus female were 370 vs 44 or 101 vs 17 in NAs naïve or in NAs treated group, respectively. The percentage of males and females were 88.5% and 11.5%. There were 55 or 137 in A or B score in NAs naïve patients; whereas there were 31 or 54 in A or B score in NAs treated group, according to the BCLC staging algorithm.

**Table 1:** Demographic, clinical characteristic, biochemical characteristic and virology characteristic

Characteristic	NAs naïve (*n* = 414)	NAs treated (*n* = 118)	*p*-value
Age, (mean ± SD)	52 ± 10	55 ± 10	< 0.01
Gender			NS
Male	370 (89.37%)	101 (85.59%)	
Female	44 (10.63%)	17 (14.41%)	
BCLC Score			< 0.001
A	55 (13.29%)	31 (26.27%)	
B	137 (33.09%)	54 (45.76%)	
C	138 (33.33%)	20 (16.95%)	
D	84 (20.29%)	13 (11.02%)	
cirrhosis			NS
No	20 (4.83%)	4 (3.39%)	
Yes	394 (95.17%)	114 (96.61%)	
Family history of HBV			NS
No	299 (72.22%)	86 (72.88%)	
Yes	115 (27.78%)	32 (27.12%)	
Family history of HCC			NS
No	370 (89.37%)	105 (88.98%)	
Yes	44 (10.63%)	13 (11.02%)	
AFP (ng/ml), median (range)	223.3 (11.3–3875.3)	64.77 (8.22–397.06)	< 0.001
ALT (IU/L), median (range)	44 (31–68)	32 (25–47)	< 0.001
AST (IU/L), median (range)	54 (35–107)	35 (26–62)	< 0.001
Total bilirubin (μmol/L), median (range)	22.6 (16.30–41.7)	20.6 (15.8–34.5)	NS
Direct bilirubin (μmol/L), median (range)	5.4 (2.9–13.6)	4 (2.8–8.1)	< 0.05
HBeAg			NS
Negative	273 (67.57%)	77 (67.54%)	
Positive	131 (32.43%)	37 (32.46%)	
HBV DNA, copies/ml			
< 10^4^	152 (42.82%)	91 (79.82%)	< 0.001
≥ 10^4^	203(57.18%)	23 (20.18%)	

BCLC Score, Barcelona Clinic liver Score; AFP, α-fetoprotein; ALT, Aspartate Aminotrasferase; AST, Aspertate Aminotransferase; HBeAg, hepatitis B e antigen; SD, standard deviation.

In NAs naïve or NAs treated groups, 394 out of 414 or 114 out of 118 CHB patients developed cirrhosis at the time of HCC diagnosed, respectively. The average level of serum AFP, ALT, AST or direct bilirubin from the NAs naïve group was 3.4-fold (*p* < 0.001), 1.4-fold (*p* < 0.001) 1.5-fold (*p* < 0.001) or 1.4-fold (*p* < 0.05) higher than that from the NAs treated group, respectively

However, there was no significant different total bilirubin between NAs naïve or NAs treated patients. Among 414 NAs naïve or 118 NAs treated HCC patients, 131 or 37 of them were HBeAg^+^ at the time of HCC diagnosis. Among 414 NAs naïve or 118 NAs treated HCC patients, 203 or 23 of them had HBV DNA ≥ 10^4^ copies/ml at the time of HCC diagnosis. Finally, among 414 NAs naïve or 118 NAs treated HCC patients, 44 or 13 patients had family history of HCC, and 115 or 32 patients had family history of HBV, respectively (Table 1).

### Overall survival in CHB-related HCC patients

All CHB-related HCC patients were followed-up the overall survival post HCC treatment. CHB-related HCC patients (*n* = 532) were divided into three groups: NAs naïve (*n* = 156), NAs post-HCC (*n* = 258) was referred as NAs initiated after HCC treatment; and sustained NAs group (*n* = 118) was referred as continuous NAs treatment prior to and post-HCC treatment (Table 2). The mean age in NAs naïve NAs post-HCC or sustained NAs was 55, 51 or 55, respectively.

**Table 2:** Demographic, clinical characteristic, biochemical characteristic

Characteristic	NAs naive (*n* = 156)	NAs post-HCC (*n* = 258)	Sustained NAs (*n* = 118)	*p*-value
Age, (mean ± SD)	55 ± 11	51 ± 10	55 ± 10	< 0.001
Gender				NS
Male	139 (89.10%)	231 (89.53%)	10 (85.59%)	
Female	17 (10.90%)	27 (10.47%)	17 (14.41%)	
Cirrhosis				NS
No	6 (3.85%)	14 (5.43%)	4 (3.39%)	
Yes	150 (96.15%)	244 (94.57%)	114 (96.61%)	
HBeAg				
Negative	114 (76.51%)	159 (62.35%)	77 (67.54%)	0.01
Positive	35 (23.49%)	96 (37.65%)	37 (32.46%)	
HBV DNA, copies/ml				
< 10^4^	59 (52.21%)	93 (38.43%)	91 (79.82%)	< 0.001
≥ 10^4^	54 (47.79%)	149 (61.57%)	23 (20.18%)	
Median Survival Days	534	874	1223	< 0.05

BCLC Score, Barcelona Clinic Liver Score; HBeAg, hepatitis B e antigen; SD, standard deviation.

**Figure 1:**
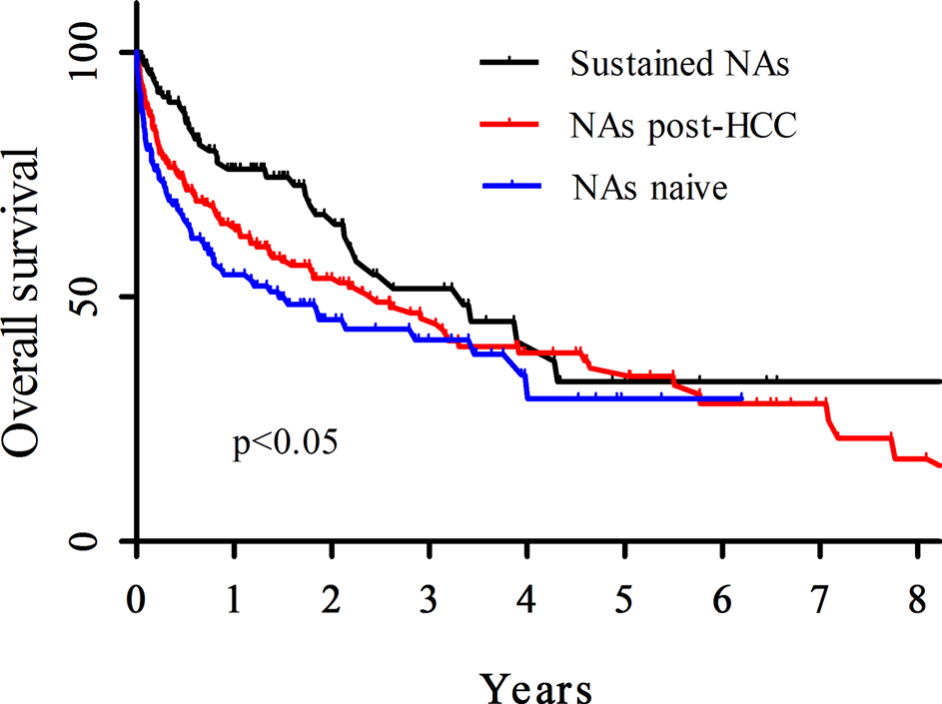
Median overall survival rate among the HCC patients This graph represented median overall survival rate among the HCC patients, using Kaplan-Meier curve analysis, the patients with NAs naïve (red), NAs post HCC (blue) or sustained NAs (black). X-axis represented years, Y-axis represented survival percent.

**Figure 2:**
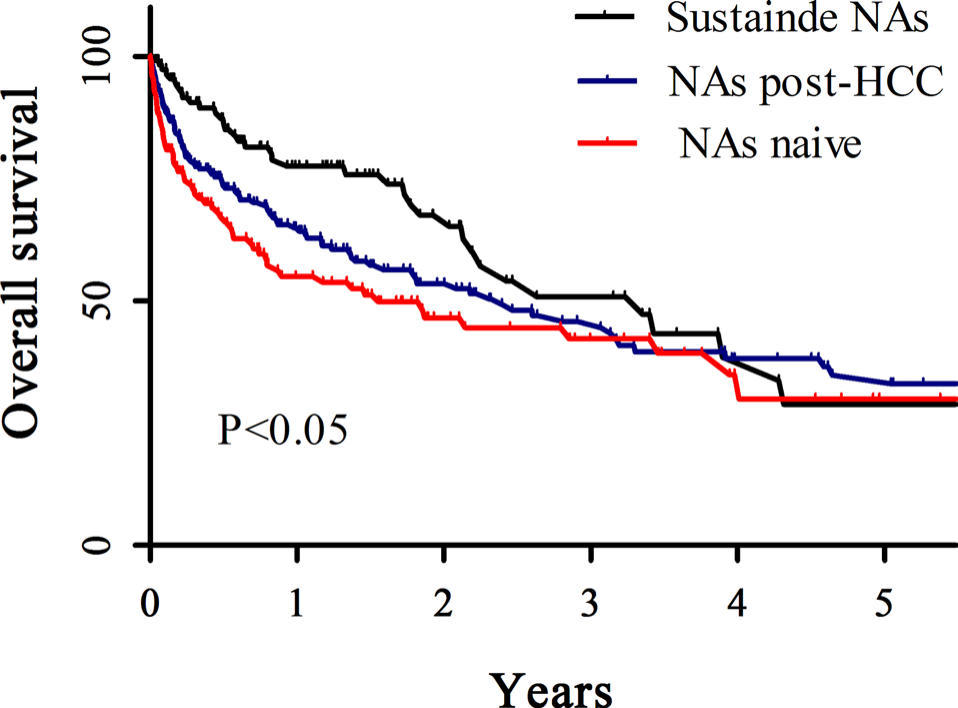
A comparison of survival rate among the cirrhosis patients at the time of diagnosis of HCC, using Kaplan-Meier curve The survival rate of the patients with NAs naïve (red), NAs post HCC (blue) or sustained NAs (black). X-axis represented years, Y-axis represented survival percent.

Among these three groups there was no significant different in gender at the time of HCC diagnosis. Out of 156 NAs naïve, 258 NAs post-HCC or 118 sustained NAs groups, there were 35, 96 or 37 CHB-related HCC patients with HBeAg^+^, respectively. There were 59, 93 or 91 patients with HBV DNA < 10^4^ copies/ml out of 156 NAs naïve, 258 NAs post-HCC or 118 sustained NAs groups, respectively (Table 2). The median survival period in the NAs naïve, NAs post-HCC or sustained NAs group was 534, 874 or 1223 days, respectively (*p* < 0.05) (Table 1). The overall survival in sustained NAs group was significantly higher than NAs post-HCC or NAs naïve groups (*p* < 0.05) (Figure 1). In addition, it was observed higher overall survival in NAs post-HCC group than NAs naïve group, although no significance (Figure 1). At the time of diagnosed HCC, 150 out of 156 NAs naïve patients were cirrhosis, but 244 out of 258 NAs post-HCC patients or 114 out of 118 sustained NAs patients were cirrhosis (Table 2). The overall survival in the sustained NAs group was also significantly higher than the NAs naïve group in the cirrhotic patients (*p* < 0.05) (Figure 2).

Among 532 CHB-related HCC patients, only 469 had exact HBV DNA records at the time of diagnosed HCC, who were further divided into low viral load group (HBV DNA < 10^4^ copies/ml) (*n* = 243) and high viral load group (HBV DNA ≥ 10^4^ copies/ml) (*n* = 226) (Table 3). The mean age in low viral load or high viral load groups was 54 or 52, respectively. Female versus male were 213 out of 243 CHB-related HCC patients with low viral load or 205 out of 226 high viral load CHB-related HCC patients, respectively.

**Table 3:** Demographic, clinical characteristic and biochemical characteristic

Characteristic	Low viral (*n* = 243)	High viral (*n* = 226)	*p*-value
Age, (mean ± SD)	54 ± 10	52 ± 10	NS
Gender			NS
Male	213 (87.65%)	205 (90.71%)	
Female	30 (12.35%)	21 (9.29%)	
Family history of HBV			
No	189 (77.78%)	147 (65.04%)	< 0.01
Yes	54 (22.22%)	79 (34.96%)	
HBeAg			
Negative	188 (78.01%)	120 (53.57%)	< 0.001
Positive	53 (21.99%)	104 (46.43%)	
NAs post-HCC			NS
Yes	184 (75.72%)	172 (76.11%)	
No	59 (24.28%)	54 (23.89%)	
Median Survival, day	1144	496	< 0.001

BCLC Score, Barcelona Clinic liver Score; HBeAg, hepatitis B e antigen; SD, standard deviation.

**Figure 3:**
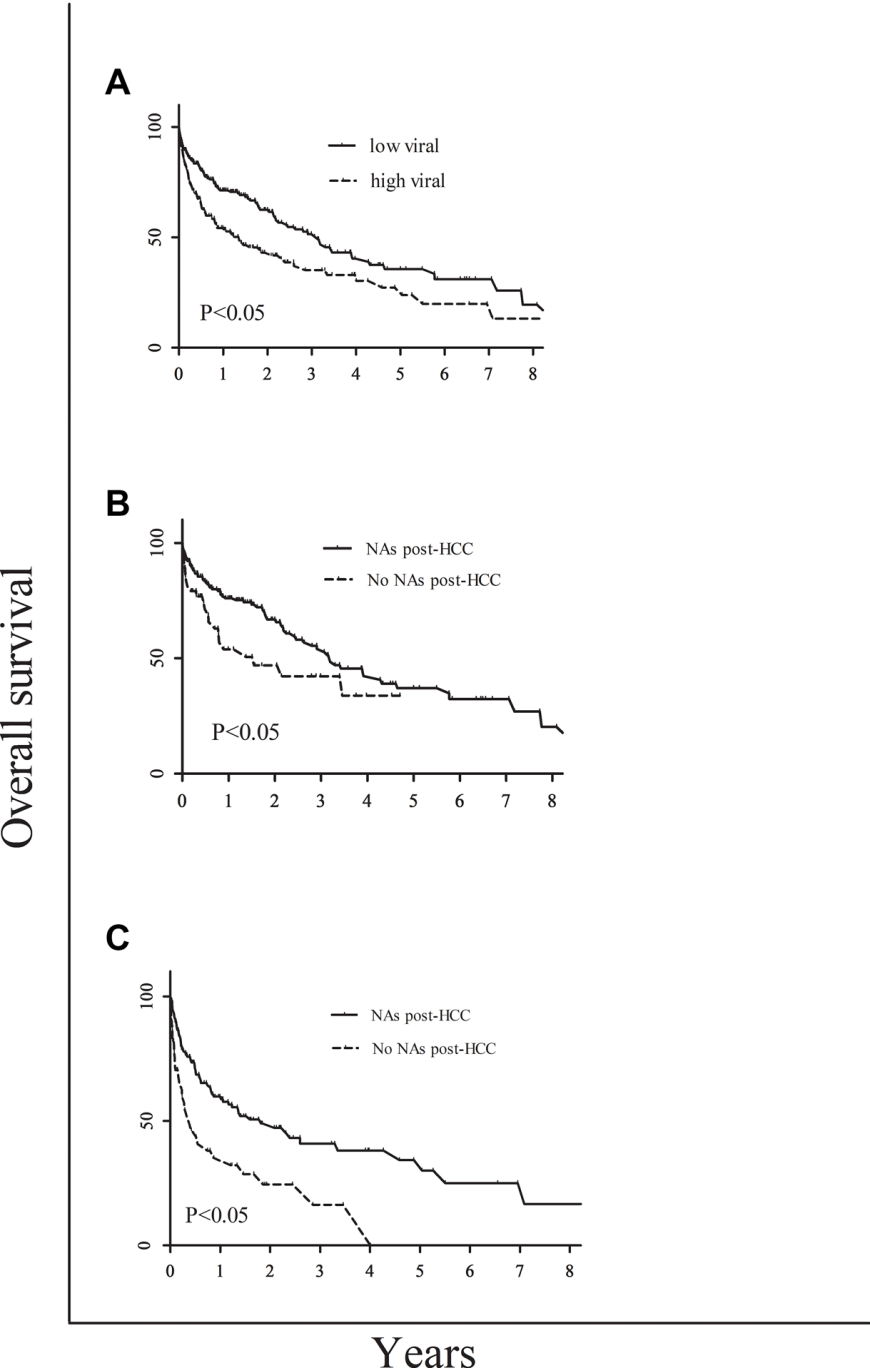
Comparison among different groups as follows Comparison of survival rate between high and low viral load in these HCC patients (**A**); comparison of the overall survival between the NAs post-HCC and no NAs post-HCC of CHB-related HCC in the high viral load group (**B**); and comparison of the overall survival between the NAs post-HCC and no NAs post-HCC of CHB-related HCC in the low viral load group (**C**).

There were 54/243 or 79/226 CHB-related HCC patients with family history of HBV with low or high viral load group, respectively. At the time of HCC diagnosis, among 53/243 or 79/226 CHB-related HCC were HBeAg^+^ with low or high viral load. The median survival in the CHB-related HCC patients with low or high viral load was 1144 or 496 days, respectively (*P* < 0.001) (Table 3). Thus the overall survival period of CHB-related HCC patients with low viral load was significantly longer than that with high viral load patients (*p* < 0.05) (Figure 3A).

There were 184 out of 243 low viral load CHB-related HCC patients had NAs post-HCC treatment but only 59 patients didn't have NAs post-HCC treatment. Furthermore, there were 172 out of 226 high viral load CHB-related HCC patients had NAs post-HCC treatment, but 54 patients didn't have NAs post-HCC treatment (Table 3). Interestingly, NAs post-HCC treatment improved the survival of both low and high viral load CHB-related HCC patients, compared to that without NAs (*p* < 0.05) (Figure 3B and 3C).

### Predictors for poor overall survival of CHB-related HCC patients

Using univariate analysis, predictors for poor overall survival of CHB-related HCC patients included: advanced BCLC stage (hazard ratio, 2.04 [95% confidence interval, 1.76–2.36], *p* < 0.001), NAs post-HCC or NAs naïve (1.36 [1.12–1.65], *P* < 0.01), serum AFP ≥ 400 ng/ml (1.34 [1.02–1.75], *P* < 0.05), serum ALT ≥ 64 IU/L (1.66 [1.24–2.21], *P* < 0.01), serum AST ≥ 40 IU/L, (2.5 [1.84–3.42], *P* < 0.001), total bilirubin ≥ 24 μmol/L (2.01 [1.54–2.63], *P* < 0.001), direct bilirubin ≥ 6.8 μmol/L (2.46 [1.89–3.21], *P* < 0.001) and HBV DNA ≥ 10^4^ copies/ml (1.64 [1.24–2.16], *P* < 0.001) (Table 4). To eliminate the interference among the predictors stated above, multivariate analysis was applied to identify the significant predictors of the poor overall survival in HCC patients. The independent predictors included: advanced BCLC stage (1.84 [1.57–2.15], *P* < 0.001), NAs post-HCC or NAs naïve (1.33 [1.07–1.65], *P* < 0.01), serum AST ≥40 IU/L (1.48 [1.03–2.12], *P* < 0.05) and HBV DNA ≥10^4^ copies/ml (1.36 [1.01–1.83], *P* < 0.001) (Table 4).

**Table 4:** Clinical and biochemical characteristics in HCC patients: Univariate and multivariate survival analysis

	OS Univariate analysis	*p*-value	Multivariate analysis	*p*-value
	HR (95% CI)		HR (95% CI)	
age	1.00 (0.99–1.01)	NS		
Sex: male/female	0.91 (0.62–1.33)	NS		
BCLC: A/B/C/D	2.04 (1.76–2.36)	< 0.001	1.84 (1.57–2.15)	< 0.001
sustained NAs/NAs post-HCC/NAs naive	1.36 (1.12–1.65)	< 0.01	1.33 (1.07–1.65)	< 0.01
AFP: < 400/≥ 400 (ng/ml)	1.34 (1.02–1.75)	< 0.05		
ALT: < 64/≥ 64 (IU/L)	1.66 (1.24–2.21)	< 0.001		
AST: < 40/≥ 40 (IU/L)	2.5 (1.84–3.42)	< 0.001	1.48 (1.03–2.12)	< 0.05
Total bilirubin: < 24/≥ 24 (μmol/L)	2.01 (1.54–2.63)	< 0.001		
Direct bilirubin: < 6.8/≥ 6.8 (μmol/L)	2.46 (1.89–3.21)	< 0.001		
HBeAg: Negative/positive	1.12 (0.85–1.48)	NS		
HBV DNA: < 10^4^/≥ 10^4^ copies/ml	1.64 (1.24–2.16)	< 0.001	1.36 (1.01–1.83)	< 0.001

BCLC Score, Barcelona Clinic liver Score; AFP, α-fetoprotein; ALT, Aspartate Aminotrasferase; AST, Aspertate Aminotransferase; HBeAg, hepatitis B e antigen.

## DISCUSSION

Our current study demonstrated that NAs reduced severity and/or progression of CHB-related HCC patients significantly, according to BCLC score and the biochemical information. In addition, CHB and post-HCC treatment with NAs contributed to improve the survival period in CHB-related HCC patients.

Nearly one quarter of CHB-related HCC patients experienced NAs (118 out of 532). In addition, the majority (~80%) of patients were NA naïve prior to HCC diagnosis among these CHB-related HCC patients with HBV family history (Table 1), suggesting that NAs were not routinely used in those CHB-related HCC patients in Ruijin Hospital, China. The guideline for CHB treatment focuses on using the antiviral therapy and other issues in 2016 [21]. Furthermore, the BCLC scores as well as ALT/AST levels and viral load in NAs treated CHB-related HCC patients was significantly better than that NAs naïve, suggesting that NAs significantly reduced the severity of CHB-related HCC. Our data is supported by previous research that NAs treatment reduces the incidence of CHB-related HCC significantly [17, 22, 23]. It has been reported that reduced viral load (< 10^4^ copies/ml) [24, 25] or pre-operational low viral load (< 10^4^ copies/ml) [26–28] extends overall survival of CHB-related HCC patients. Our current findings further identify that the overall survival of CHB-related HCC patients with low viral load was significantly higher than these patients with high viral load at the time of HCC diagnosis.

Low viral load CHB-related HCC patients with NAs therapy following HCC treatment has therefore been identified to significantly improve survival period compared to patients without NAs. Such data is supported by the study of NAs on post-operative or liver transplantation prognosis of CHB-related HCC patients [24, 29, 30]. Our data showed that the overall survival of HBV related HCC patients with sustained NAs was substantially better than NAs imitated post-HCC or NAs naïve HCC patients. There was no significant different overall survival between NAs post-HCC and NAs naïve HCC patients. In addition, among HBV related HCC cirrhosis patients, the overall survival in the sustained NAs group was also significantly better than the NAs naïve group. Interestingly, multivariate analysis revealed advanced BCLC stage, post-HCC with or without NAs, serum AST ≥ 40 IU/L, HBV DNA ≥ 10^4^ copies/ml were independent predictors for overall survival of HCC patients. Importantly, sustained NAs in CHB-related HCC patients retained independent prognosis power under the multivariate analysis.

Limitations to this study include the restricted patient population range, specifically the CHB-related HCC patients from the Ruijin Hospital, Shanghai, China, were from relatively closed regional near Shanghai (eastern China). In future study we will collaborate with our colleagues from different regions in China, which will offer even more objective demographic. Second, options for HCC treatments were not taken into consideration, which we will analysis the influence among different NAs in reducing severity of CHB-related HCC. In addition, we will analyze the difference of survival between chemotherapy and surgery for CHB-related HCC patients. Identification of the relationship between other aspects of method of treatment of HCC (e.g. surgery, other forms of chemotherapy) have not been analysed, due to the relative small numbers of the samples, which will be determined in future. Finally, we will determine the possible mechanism of HCC patients developed directly from CHB patients without cirrhosis.

In conclusion, NAs reduced substantially the severity of CHB-related HCC patients, despite a small number of NA treated CHB patients still developed into HCC. NAs significant extended survival period post-HCC treatment, which is supported by the study of NAs on post-operative or liver transplantation prognosis of CHB-related HCC patients with low viral load HCC patients had a significant better prognosis than that with high viral load [31]. Survival period in CHB-related HCC patients with NAs sustained was extended significantly.

## MATERIALS AND METHODS

### Patients and study design

**Figure 4:**
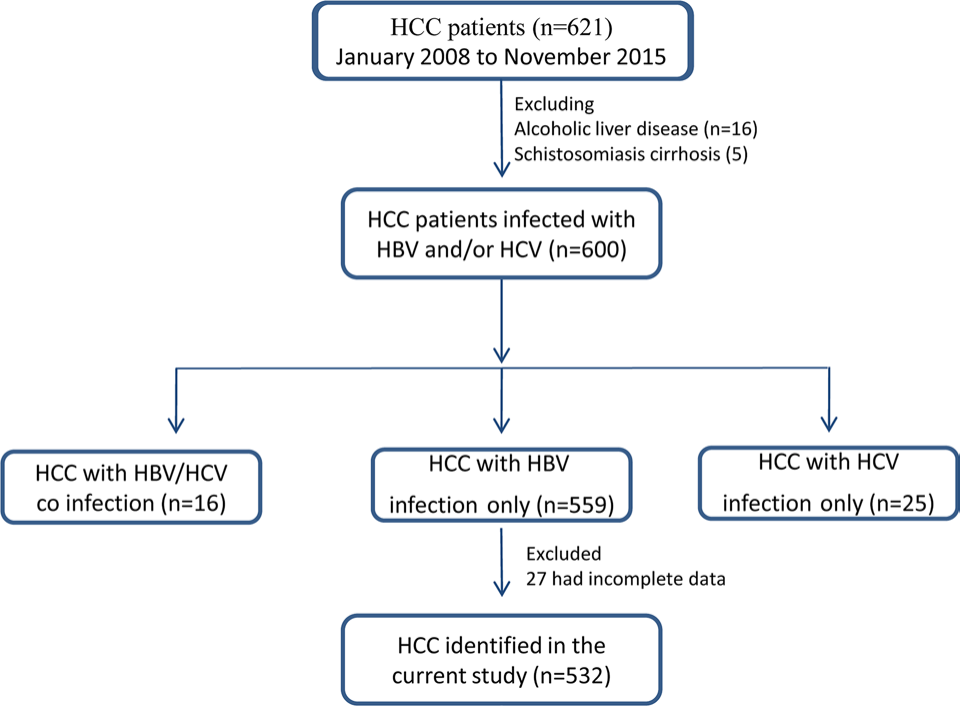
The diagram illustrated the recruitment and selection of patients for the current study

A single-centre retrospective study was designed by screening HCC patients in the Ruijin Hospital (Shanghai, China) from January 1, 2008 to November 1, 2015. Inclusion criterions were: HCC patients with HBV infection; complete clinical characteristic and laboratory data at the time of diagnosis of HCC. The exclusion criteria were: HCC caused by HCV, alcoholic liver disease or schistosomiasis cirrhosis; patients with extra-hepatic malignancy; patients with incomplete data. A total of 621 HCC patients were identified initially from the hospital database and 89 of them were subsequently excluded, because: 1) alcoholic liver disease (*n* = 16); 2) schistosomiasis cirrhosis (*n* = 5), 3) HCV infection (*n* = 25), 4) HBV and HCV co-infection (*n* = 16), 5) incomplete data (*n* = 27). The finally eligible CHB-related HCC patients were 532 (Figure 4).

This study complies with the Declaration of Helsinki, and has been approved by *the Human Ethics Committee, Rui-Jin Hospital, Shanghai Jiao Tong University School of Medicine*.

### Diagnosis

All suspected patients underwent laboratory examinations, including HBsAg, HBeAg, Anti-HCV, ALT, AST, total bilirubin, direct bilirubin and α-fetoprotein. Imaging procedures (abdominal ultrasonography, CT, MRI) were regularly performed to identify imaging evidence of HCC. Liver biopsy was performed in some of the patients to confirm the presence of HCC. The diagnosis of HCC was based on clinical, serological, radiological and/or histological evidence [32]. Barcelona Clinic Liver Cancer (BCLC) score [33] was applied for each patient at enrollment time to optimize therapeutic strategy and to predict prognosis.

### Data collection

In this study, the basic information was collected, including sex, age at the same time of diagnosis HCC, family history of HBV and family history of HCC. The information of CHB-related HCC with NAs treatment at the CHB stage was critical. Dosage and treatment period of NAs were recorded in those CHB-related HCC patients. It was confirmed if there was cirrhosis at the same time of diagnosis HCC. Moreover, it was identified whether HCC patients with cirrhosis at the time of initial NAs for the HCC patients infected HBV. Cirrhosis was diagnosed according to liver biopsy or the portal hypertension, esophageal gastric varices, splenomegaly, ascites depending on the image diagnosis (abdominal ultrasonography, CT and MRI).

The laboratory data, identified at the same time of the patients diagnosed HCC, included: liver function tests (ALT/AST/total bilirubin/direct bilirubin), a-fetoprotein (AFP), HBeAg and HBV DNA load. The level of AFP from HCC patients was divided into AFP < 400 ng/ml and AFP ≥ 400 ng/ml. The abnormal levels of the laboratory data were ALT <64 IU/L, AST < 40 IU/L, total bilirubin < 24 μmol/L and direct bilirubin < 6.8 μmol/L. We defined the HBV DNA levels of ≥ 10^4^ copies/ml as high viral load and HBV DNA levels of < 10^4^ copies/ml as low viral load, as described previously 12.

### Follow-up

In this study, the follow-up information for 532 HCC patients included the overall survival and NAs post HCC treatment. The average of follow-up time was 211 days.

### Statistics

Student's *t* test was used to compare the continuous variables with normal distributions. The variables without normal distributions were studied by the Wilcoxon signed-rank test. Chi-square or Fisher's exact test was employed to study the categorical variables. The univariate analysis for different clinical factors associated with the poor overall survival of HCC patients were used the Kaplan-Meier statistics and Log-rank test. The Cox stepwise selection regression analysis was used to assess the impact of the different factors associated with the poor overall survival on the multivariate analysis. The variables with a *p*-value < 0.05 entered the Cox stepwise selection regression model at each step of the multivariate analysis, whereas the variables were removed from the model with the *p*-value > 0.05. The *p*-value < 0.05 was taken to be significant in all the other statistical. The SAS 9.4 software was used to perform the data analysis. The Figures were made using GraphPad Prism 5 software. Continuous variables with normal distributions expressed means and means of SD. Continuous variables without normal distributions are expressed as medians (range).
